# Postoperative chylothorax following congenital heart surgery: Incidence, risks, therapy, and possible mechanisms

**DOI:** 10.1016/j.xjon.2026.102062

**Published:** 2026-08-18

**Authors:** Thibault Schaeffer, Verena Kunke, Jonas Palm, Andrea Amici, Teresa Lemmen, Christina Ruda, Nicole Piber, Muneaki Matsubara, Paul Philipp Heinisch, Alfred Hager, Peter Ewert, Jürgen Hörer, Masamichi Ono

**Affiliations:** a Department of Congenital and Pediatric Heart Surgery, German Heart Center, TUM University Hospital, Munich, Germany; b Division of Congenital and Pediatric Heart Surgery, University Hospital of Munich, Ludwig-Maximilians-Universität, Munich, Germany; c Europäisches Kinderherzzentrum München, Munich, Germany; d Department of Congenital Heart Disease and Pediatric Cardiology, German Heart Center, TUM University Hospital, Munich, Germany; e Department of Cardiovascular Surgery, German Heart Center, TUM University Hospital, Munich, Germany

**Keywords:** postoperative chylothorax, congenital heart surgery, Fontan procedure

## Abstract

**Objective:**

To determine the incidence, risks factors, outcomes, and mechanisms of postoperative chylothorax (PCT) following congenital heart surgery.

**Methods:**

Among all patients who underwent congenital heart surgery between 2001 and 2024, those who developed PCT during the same hospitalization were identified. The prevalence of chylothorax was analyzed, along with associated procedures, risk factors, and hemodynamics.

**Results:**

Among 9892 congenital heart surgeries performed during the study period, 312 patients (3.2%) developed PCT. Patients with PCT had a higher proportion of males (64% vs 56%; *P* = .009), younger age at surgery (median, 0.6 years vs 1.2 years; *P* < .001), and lower weight at surgery (median, 6.1 kg vs 8.6 kg; *P* < .001) compared to those without PCT. Regarding surgical types, the most frequently performed operation was the Fontan procedure (20.5%), followed by repair of tetralogy of Fallot (TOF) with atrioventricular septal defect (11.8%), arterial switch operation (ASO), ventricular septal defect (VSD) closure, and aortic arch repair (10.0%). Younger age at operation (*P* < .001; hazard ratio [HR], 0.745), the Fontan procedure (*P* < .001; HR, 16.198), ASO, VSD closure, and aortic arch repair (*P* < .001; HR, 2.835) were strong risk factors for PCT. Postoperative lower mean arterial pressure and higher central venous pressure were associated with PCT in patients following the Fontan procedure and TOF repair.

**Conclusions:**

This large, single-center study of approximately 10,000 congenital heart surgeries reported a 3.2% incidence of PCT. The Fontan procedure had the highest incidence of PCT, followed by complex biventricular repair in neonates and young infants. Early postoperative lower mean arterial pressure and higher central venous pressure may trigger PCT.

## Graphical Abstract

Graphical abstract summarizing study design, key findings, and clinical implications.

**Figure:**
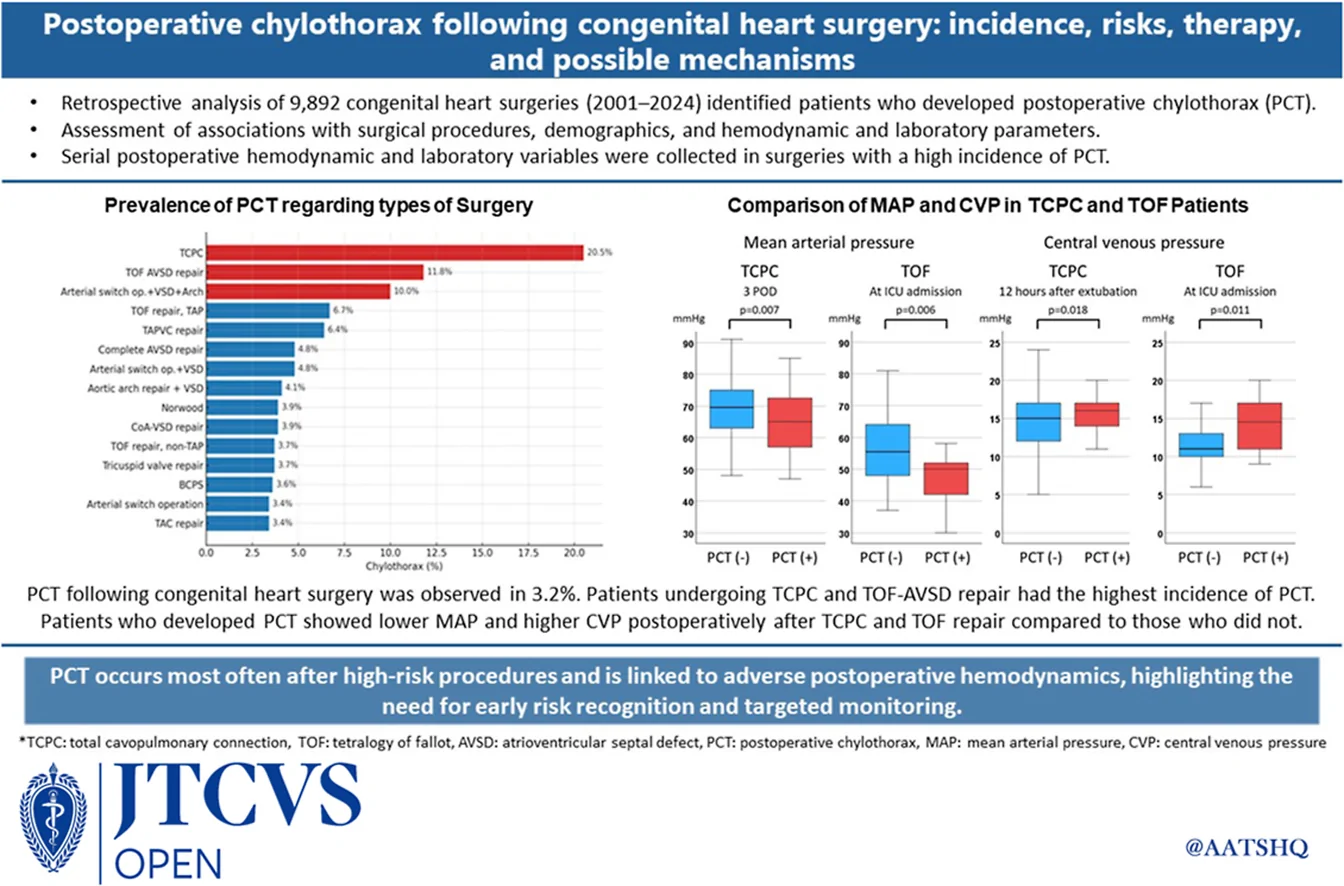

**Figure:**
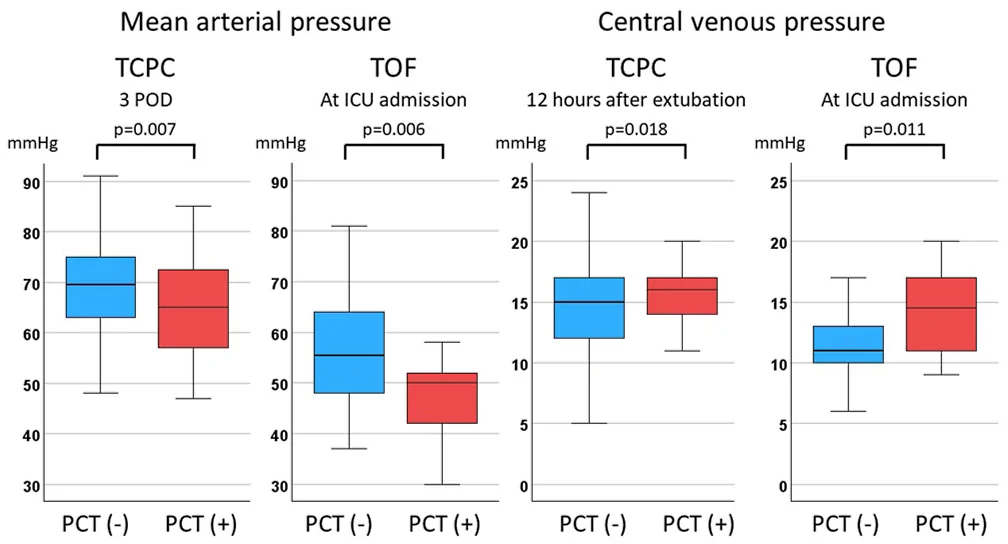
Mean arterial pressure and central venous pressure in patients with TCPC and TOF.

**Central Message**

The incidence of postoperative chylothorax was 3.2% after 9892 congenital heart surgeries. Unstable hemodynamics in the early postoperative phase may be a major cause of subsequent chylothorax.

**Perspective**

Postoperative chylothorax (PCT) was observed in 3.2% of patients. The Fontan procedure and complex biventricular repair in neonates and young infants had the highest incidence. Strategies should focus on addressing early postoperative hemodynamic instability to prevent PCT.

The incidence of postoperative chylothorax (PCT) after congenital heart surgery ranges from 1% to 8%, depending on patient age and surgical type.^1^, ^2^, ^3^, ^4^ Not only mechanical trauma to lymphatic vessels and hemodynamic disturbances but also underlying congenital lymphatic malformations, such as pulmonary lymphatic perfusion syndrome (PLPS) and central lymphatic flow disorder (CLFD), are known to be responsible for PCT.^2^^,^^3^ Because this complication is associated with significant hospital morbidity, mortality, and cost, a proper diagnosis and appropriate treatment are crucial; however, definitive guidelines have not yet been established,^4^ making it important to evaluate the contemporary incidence of PCT stratified by procedure and to identify associated risk factors.

A deeper understanding of the exact mechanisms underlying PCT could pave the way for targeted preventive strategies and earlier recovery, ultimately reducing the incidence and burden of this complication in future clinical practice. Studies examining PCT after congenital heart surgery remain limited, however,^1^, ^2^, ^3^, ^4^, ^5^, ^6^, ^7^, ^8^, ^9^ and information on risk factors is insufficient.^8^^,^^9^ Furthermore, assessment of hemodynamic data for the development of PCT has not been fully performed. Regarding management, several therapeutic options are available, including dietary modifications, medications, and surgery.^10^, ^11^, ^12^, ^13^, ^14^, ^15^, ^16^, ^17^ Recent developments in magnetic resonance (MR) lymphangiography provide new opportunities to optimize diagnostic and therapeutic strategies for PCT.^3^^,^^18^

The present study aimed to determine the incidence, risk factors, and treatment of PCT after congenital heart surgery using our large contemporary cohort and to evaluate its impact on postoperative hospital stay and mortality. Furthermore, we performed hemodynamic assessment in procedures with a high incidence of PCT and aimed to clarify the clinical characteristics of patients who developed PCT.

## Methods

### Data Availability Statement

The data that support the findings of this study are available from the corresponding author on reasonable request.

### Ethical Statement

This study was approved by the Institutional Review Board of the Technical University of Munich (approval 2025-232-S-NP, approved May 5, 2025). Because of the study’s retrospective nature, the need for individual patient consent was waived.

### Patients and Data Collection

This single-center study analyzed hospitalizations among all patients who underwent congenital heart surgery with cardiopulmonary bypass (CPB) at TUM University Hospital, German Heart Center, between 2001 and 2024. Surgery was performed using standard CPB with bicaval venous cannulation and arterial return to the ascending aorta. Before initiating CPB, subtotal thymus removal was performed in neonates and infants, partial removal in children, and no removal in adults. For the Fontan procedure, we cannulated both the superior and inferior vena cava directly and snared the venous cannula to establish total CPB. We then incised the right pulmonary artery and performed a distal anastomosis with the extracardiac conduit. Regarding control of the previous systemic-to-pulmonary shunt, we usually terminated the systemic-to-pulmonary flow at the time of stage II bidirectional cavopulmonary shunt (BCPS). A 4.5 Fr central venous catheter was used for operations in neonates and young infants. Postoperative thromboprophylaxis was standardized for all patients. Unfractionated heparin was administered intravenously at a dose of 5000 IU/m^2^/d. The target partial thromboplastin time was set at 60 seconds. This regimen was maintained until all central lines were removed.

Medical records were collected, including baseline morphology and demographics, as well as periprocedural data, and patients who developed PCT were identified. Age was classified into the following groups according to age at admission: neonates (≤30 days), infants (≥31 days and <1 year), young children (≥1 year and <6 years), older children (≥6 years and <12 years), teenagers (≥12 years and <18 years), and adults (≥18 years). Procedural risk was categorized using the Society of Thoracic Surgeons–European Association for Cardio-Thoracic Surgery (STAT) score. Era analysis was performed comparing the early era (2001-2012) and late era (2013-2024).

### Definition of PCT and Treatment

PCT was defined as a condition in which lymphatic fluid was found in the thoracic drainage postoperatively. Pleural fluid in patients with persistent postoperative chest tube drainage >5 mL/kg/day was routinely tested for triglycerides, leukocytes, and lymphocyte fraction in the pleural fluid. Our major diagnostic criteria for chylothorax were triglycerides >100 mg/dL, leukocytes >1000/μL, and a lymphocyte fraction >80%. Routine detection of chylomicrons in the pleural fluid via lipoprotein electrophoresis was not performed. The first-line medical treatment for confirmed chylothorax consisted of fat restriction (1 g per 100 g meal) or oral substitution with a medium-chain triglyceride (MCT) formula. Total parenteral nutrition (TPN) was provided to selected patients, and intravenous infusion of octreotide (3-15 μ/kg/hour) was performed in patients who had no pathway obstructions, confirmed by cardiac catheterization. If a patient did not respond to conservative treatment, cardiac catheterization was performed to rule out any anatomic issues. Thoracic duct ligation was considered when 2 cycles of medical treatment failed, and no anatomic lesions were detected on cardiac catheterization. MR lymphangiography was performed in selected patients to confirm the congenital lymphatic malformations, such as PLPS and CLFD.

### Hemodynamic Assessment in Surgeries With a High Incidence of PCT

Serial postoperative hemodynamic and laboratory variables were collected for the surgeries with a high incidence of PCT using an electronic intensive care unit (ICU) chart. Variables included postoperative mean arterial pressure, central venous pressure, arterial oxygen saturation, and ICU lactate levels.

### Statistical Analysis

Categorical variables were presented as absolute number and percentage and compared using the χ^2^ test. Continuous variables are expressed as median with interquartile range (IQR). Comparisons of groups were done using the independent-sample Student *t* test for normally distributed variables and the Mann-Whitney *U* test for non-normally distributed variables. The association between preoperative factors and PCT was explored using logistic regression and reported as odds ratio (OR) with 95% confidence interval (CI). Data analysis was performed using SPSS version 28.0 for Windows (IBM) and R version 4.3.3 (R Foundation for Statistical Computing).

## Results

### Patient Characteristics and Perioperative Data

Among the 9892 procedures performed during the study period, 312 patients (3.2%) developed PCT. Patient characteristics are shown in Table 1. PCT was observed more frequently in neonates (*P* = .045) and younger children (*P* < .001) and less frequently in older children (*P* < .001), teenagers (*P* < .001), and adults (*P* < .001). PCT developed less frequently in patients in STAT category 1 (*P* < .001), and more frequently in patients in STAT category 2 (*P* < .001). There was no significant difference associated with other STAT categories. As for perioperative and postoperative variables, patients who developed PCT required less aortic cross-clamping (*P* = .011). In patients with PCT, pleural fluid examination showed a median triglyceride level of 264 (IQR, 143-703) mg/dL, a median leukocyte count of 2000 (IQR, 800-5200)/μL, and a median lymphocyte fraction of 92% (IQR, 78%-98%). Baseline and operative characteristics of patients by era are listed in Table E1. PCT occurred significantly more often in the late era compared to the early era (4.3% vs 2.1%; *P* < .001). However, patients in the late era were significantly younger at the time of operation (*P* < .001), had longer CPB times (*P* < .001), and more frequently required aortic cross-clamping (*P* < .001).

**Table 1:** Baseline and operative characteristics of patients with and without PCT

Variable	All patients	PCT (−)	PCT (+)	*P* value
Number of patients (%)	9892	9580 (96.8)	312 (3.2)	
Preoperative variables, n (%)				
Male sex	5567 (56.3)	5369 (56.0)	198 (63.5)	**.009**
Genetic anomalies	1384 (14.5)	1338 (14.5)	46 (14.8)	.898
Extracardiac anomalies	1721 (18.1)	1662 (18.1)	59 (19.0)	.687
Heterotaxy syndrome	211 (2.2)	201 (2.2)	10 (3.2)	.232
Hypothyroidism	237 (2.5)	226 (2.5)	11 (3.5)	.235
Early era, 2001-2012	5153 (52.1)	5047 (52.7)	106 (34.0)	**<.001**
Late era, 2013-2024	4739 (47.9)	4533 (47.3)	206 (66.0)	
Operative variables				
Age at procedure, y, median (IQR)	1.2 (0.3-9.8)	1.2 (0.3-10.4)	0.6 (0.2-2.0)	**<.001**
Weight at procedure, kg, median (IQR)	8.5 (4.5-25.6)	8.6 (4.6-28.4)	6.1 (4.0-11.0)	**<.001**
OP in neonates, n (%)	1649 (16.7)	1584 (16.5)	65 (20.8)	**.045**
OP in infants, n (%)	3125 (31.6)	3022 (31.5)	103 (33.0)	.583
OP in young children (≥1 & <6 y), n (%)	2115 (21.4)	1976 (20.6)	139 (44.6)	**<.001**
OP in older children (≥6 & <12 y), n (%)	789 (8.0)	786 (8.2)	3 (1.0)	**<.001**
OP in teens (≥12 & <18 y), n (%)	699 (7.1)	698 (7.3)	1 (0.3)	**<.001**
OP in adults (≥18 y), n (%)	1515 (15.3)	1514 (15.8)	1 (0.3)	**<.001**
STAT category, n (%)				
1	4195 (42.4)	4143 (43.2)	52 (16.7)	**<.001**
2	2508 (25.4)	2342 (24.4)	166 (53.2)	**<.001**
3	1083 (10.9)	1046 (10.9)	37 (11.9)	.601
4	1682 (17.0)	1642 (17.1)	40 (12.8)	.046
5	424 (4.3)	407 (4.2)	17 (5.4)	.303
4 or 5	2106 (21.3)	2049 (21.4)	57 (18.3)	.185
CPB time, min, median (IQR)	86 (55-127)	86 (55-125)	86 (58-126)	.531
Need for aortic cross-clamp, n (%)	7278 (73.6)	7068 (73.8)	210 (67.3)	**.011**
Postoperative variables				
Pleural fluid examination, median (IQR)				
Triglycerides, mg/dL			264 (143-703)	
Leukocytes, μL			2000 (800-5200)	
Lymphocyte fraction, %			92 (78-98)	
Hospital deaths, n (%)	432 (4.4)	428 (4.5)	4 (1.3)	**.007**
Hospital length of stay, d, median (IQR)	13 (9-20)	12 (9-20)	23 (14-37)	**<.001**

Bold type indicates statistical significance. *PCT*, Postoperative chylothorax; *IQR*, interquartile range; *OP*, operation; *STAT*, Society of Thoracic Surgeons-European Association for Cardio-Thoracic Surgery; *CPB*, cardiopulmonary bypass.

### Incidence of PCT According to Type of Procedure

Procedures (n ≥15) associated with the highest rates of PCT are listed in Table 2 and Figure 1. The Fontan procedure with total cavopulmonary connection (TCPC) was the most frequently performed procedure (20.5%), followed by simultaneous repair of tetralogy of Fallot (TOF) and atrioventricular septal defect (AVSD; 11.8%). The arterial switch operation (ASO), ventricular septal defect (VSD) closure, and aortic arch repair each had a prevalence of 10.0%. Lower rates were observed after TOF repair with a transannular patch (6.7%) and after repair of total anomalous pulmonary venous connection (6.4%).

**Table 2:** High-risk procedures for PCT

Operation	Cases	Chylothorax (+), n (%)	*P* value
TCPC	584	120 (20.5)	**<.001**
TOF AVSD repair	17	2 (11.8)	**.042**
Arterial switch operation + VSD + arch	20	2 (10.0)	.080
TOF repair, TAP	240	16 (6.7)	**.002**
TAPVC repair	110	7 (6.4)	.053
Complete AVSD repair	392	19 (4.8)	.050
Arterial switch operation + VSD	105	5 (4.8)	.343
Aortic arch repair + VSD	122	5 (4.1)	.548
Norwood	408	16 (3.9)	.365
CoA-VSD repair	51	2 (3.9)	.753
TOF repair, non-TAP	215	8 (3.7)	.631
Tricuspid valve repair	191	7 (3.7)	.683
BCPS	586	21 (3.6)	.540
ASO	352	12 (3.4)	.780
TAC repair	58	2 (3.4)	.898

Bold type indicates statistical significance. *PCT*, Postoperative chylothorax; *TCPC*, total cavopulmonary connection; *TOF*, tetralogy of Fallot; *AVSD*, atrioventricular defect; *VSD*, ventricular septal defect; *TAP*, transannular patch; *TAPVC*, total anomalous pulmonary venous connection; *CoA*, coarctation of the aorta; *BCPS*, bidirectional cavopulmonary shunt; *ASO*, arterial switch operation; *TAC*, truncus arteriosus communis.

**Figure 1:**
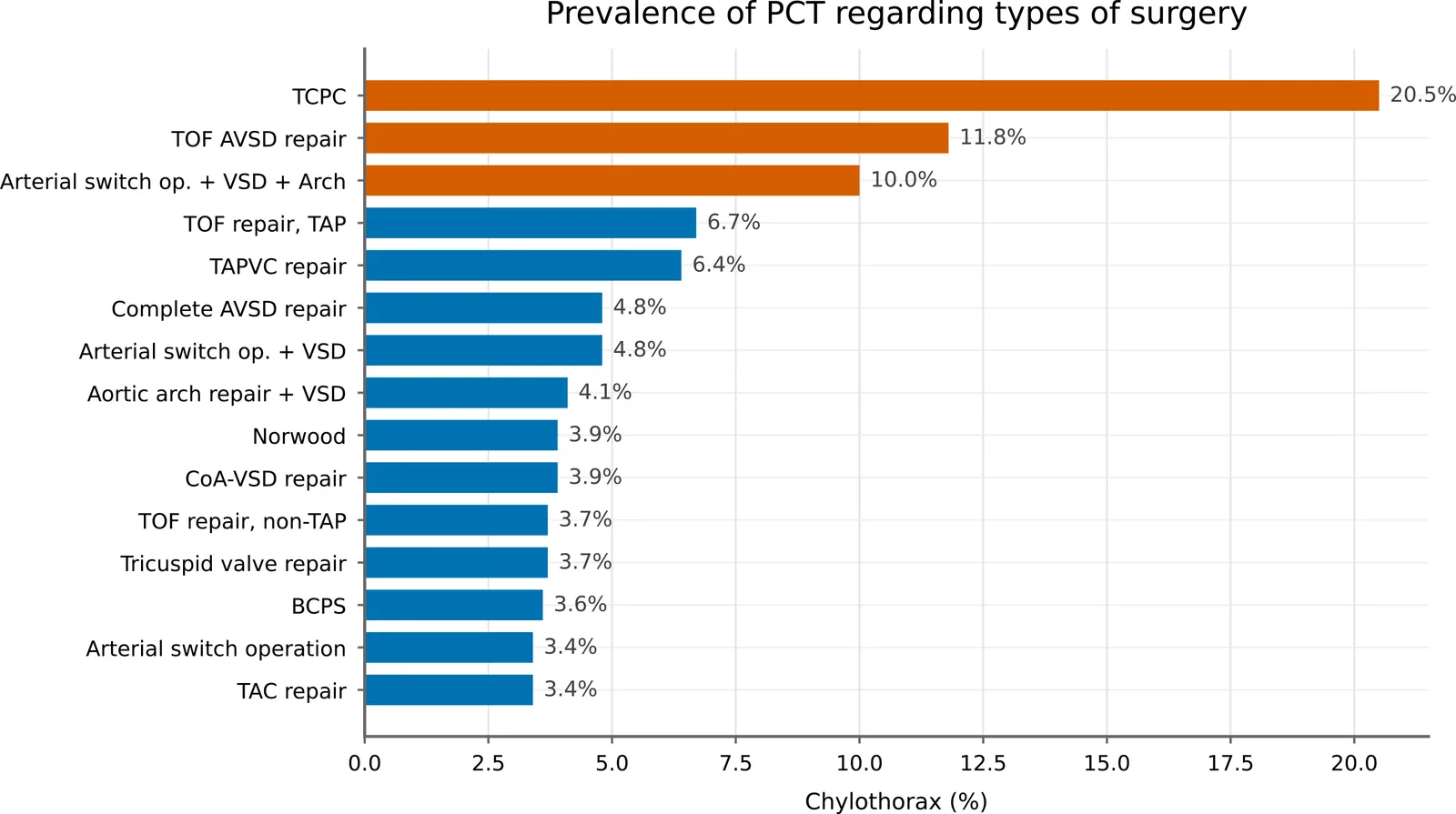
Prevalence of postoperative chylothorax (*PCT*) by surgical procedure. *TCPC*, Total cavopulmonary connection; *TOF*, tetralogy of Fallot; *AVSD*, atrioventricular defect; *VSD*, ventricular septal defect; *TAP*, transannular patch; *TAPVC*, total anomalous pulmonary venous connection; *CoA*, coarctation of the aorta; *BCPS*, bidirectional cavopulmonary shunt; *TAC*, truncus arteriosus communis.

### Risk Factors for the Development of PCT

The results of the risk factor analysis are shown in Table 3. Younger age at operation (*P* < .001; OR, 0.745), TCPC (*P* < .001; OR, 16.198), TOF-AVSD repair (*P* = .011; OR, 7.563), ASO + VSD closure + aortic arch repair (*P* < .001; hazard ratio, 2.835), TOF repair with transannular patch (*P* = .035; OR, 2.531), and complete AVSD repair (*P* = .020; OR, 1.904) were identified as independent risk factors. Genetic anomalies (*P* = .898; OR, 1.021; 95% CI, 0.743-1.404) and extracardiac anomalies (*P* = .687; OR, 1.061; 95% CI, 0.795-1.416) were not identified as significant risk factors. Stage II BCPS (*P* = .540; OR, 1.152; 95% CI, 0.733-1.808) was not identified as a risk factor.

**Table 3:** Risk factors for PCT

Variable	Univariable analysis			Multivariable analysis		
	*P* value	OR	95% CI	*P* value	OR	95% CI
Male sex	**.010**	1.362	1.078-1.721			
Age at operation	**<.001**	0.857	0.822-0.893	**<.001**	0.745	0.669-0.831
Weight at operation	**<.001**	0.949	0.939-0.964			
Operation in neonates	**.046**	1.328	1.006-1.755			
Operation in children	**<.001**	1.497	1.193-1.877			
Procedure						
TCPC	**<.001**	12.279	9.597-15.710	**<.001**	16.198	11.406-23.001
TOF-AVSD repair	.061	4.114	0.937-18.068	**.011**	7.563	1.586-36.059
ASO + VSD + arch	.099	3.427	0.792-14.835	**<.001**	2.835	1.556-5.167
TOF repair, TAP	**.002**	2.258	1.342-3.798	**.035**	2.531	1.070-5.988
TAPVC repair	.058	2.112	0.974-4.579			
Complete AVSD repair	.052	1.601	0.995-2.575	**.020**	1.904	1.109-3.269
Reoperation	**<.001**	1.850	1.474-2.322			
STAT category	**.002**	1.145	1.051-1.247			

Bold type indicates statistical significance. *PCT*, Postoperative chylothorax; *OR*, odds ratio; *CI*, confidence interval; *TCPC*, total cavopulmonary connection; *TOF*, tetralogy of Fallot; *AVSD*, atrioventricular defect; *ASO*, arterial switch operation; *VSD*, ventricular septal defect; *TAP*, transannular patch; *TAPVC*, total anomalous pulmonary venous connection; *STAT*, Society of Thoracic Surgeons–European Association for Cardio-Thoracic Surgery.

Anomalous systemic venous connections were present in 46 of the 312 patients (14.7%), including 41 with bilateral superior vena cava, 6 with azygos/hemi-azygos continuations, and 2 with anomalous hepatic venous connections. Approximately 45% (n = 20 of 46) of anomalous systemic venous connections occurred in patients undergoing a Fontan procedure. Therefore, the impact of anomalous systemic venous connections on the development of PCT was evaluated in this subset. The incidence of PCT was similar in Fontan patients with and without anomalous systemic venous connections (26.3% vs 21.6%; *P* = .360). In patients with TOF, a right aortic arch was present in 88 of 455 patients (19.3%). Previous palliation was performed in 94 patients (20.7%). The incidence of PCT was similar in patients with and without a right aortic arch (5.7% vs 5.2%; *P* = .849) and in those with and without previous palliation (4.3% vs 5.5%; *P* = .620).

### Hemodynamic Assessment in Patients With TCPC and TOF

Postoperative hemodynamic and laboratory variables were assessed in patients undergoing procedures with a high incidence of PCT (ie, TCPC and TOF). Variables in patients with TCPC are shown in Table 4. Among patients undergoing TCPC, those who developed PCT had lower mean arterial pressure 1 hour before extubation (*P* = .026), 1 hour after extubation (*P* = .016), and on postoperative day 3 (*P* = .007; Figure 2) compared to those without PCT. Central venous pressure was higher at 6 hours (*P* = .025) and at 12 hours after extubation (*P* = .018; Figure 2) in patients with PCT. Variables in patients with TOF are listed in Table 5. Patients with PCT had a lower mean arterial pressure (*P* = .006) and higher central venous pressure (*P* = .011) at ICU admission (Figure 2). Arterial oxygen saturation at ICU admission (*P* = .032) and at 6 hours after ICU admission (*P* = .027) was lower in patients with PCT (Figure 2).

**Table 4:** Hemodynamic and laboratory variables in patients with TCPC

Variables	PCT (+)		PCT (−)		*P* value
	Mean	SD	Mean	SD	
Mean arterial pressure					
At ICU admission	53.84	9.59	54.65	10.62	.578
1 h before extubation	56.60	9.47	60.07	11.48	**.026**
1 h after extubation	56.72	8.02	59.87	9.60	**.016**
6 h after extubation	59.63	11.59	61.49	11.05	.242
12 h after extubation	62.26	11.28	64.17	11.51	.244
1 POD	61.87	12.77	64.22	10.58	.136
2 POD	62.79	10.77	65.33	11.14	.105
3 POD	65.39	9.82	69.19	9.84	**.007**
Central venous pressure					
At ICU admission	16.86	3.85	17.42	3.63	.275
1 h before extubation	17.44	3.12	17.67	4.42	.646
1 h after extubation	16.03	4.04	15.82	3.98	.713
6 h after extubation	15.63	3.84	14.41	3.77	**.025**
12 h after extubation	15.92	3.17	14.64	3.95	**.018**
1 POD	15.46	4.01	14.50	3.58	.065
2 POD	13.94	3.51	14.30	3.86	.506
3 POD	14.02	3.60	14.58	3.58	.358
Arterial oxygen saturation					
At ICU admission	95.72	5.77	96.37	5.51	.411
1 h before extubation	94.74	5.95	95.38	5.07	.395
1 h after extubation	91.38	7.24	92.68	7.04	.196
6 h after extubation	92.84	5.85	94.09	6.20	.150
12 h after extubation	93.68	4.80	94.39	4.90	.314
1 POD	93.90	5.39	94.47	4.88	.419
2 POD	95.03	4.74	94.70	4.93	.635
3 POD	94.34	4.86	94.75	4.61	.538
Lactate levels					
At ICU admission	1.63	1.16	1.59	0.68	.732
1 h before extubation	2.63	1.78	2.45	1.36	.378
1 h after extubation	3.38	1.98	3.30	1.54	.737
6 h after extubation	3.91	1.76	3.78	1.85	.603
12 h after extubation	2.70	1.55	2.57	1.48	.560
1 POD	1.95	1.73	1.75	0.85	.222
2 POD	1.53	0.89	1.35	0.74	.116
3 POD	1.44	0.67	1.33	0.71	.352

Bold type indicates statistical significance. *TCPC*, Total cavopulmonary connection; *PCT*, postoperative chylothorax; *SD*, standard deviation; *ICU*, intensive care unit; *POD*, postoperative day.

**Figure 2:**
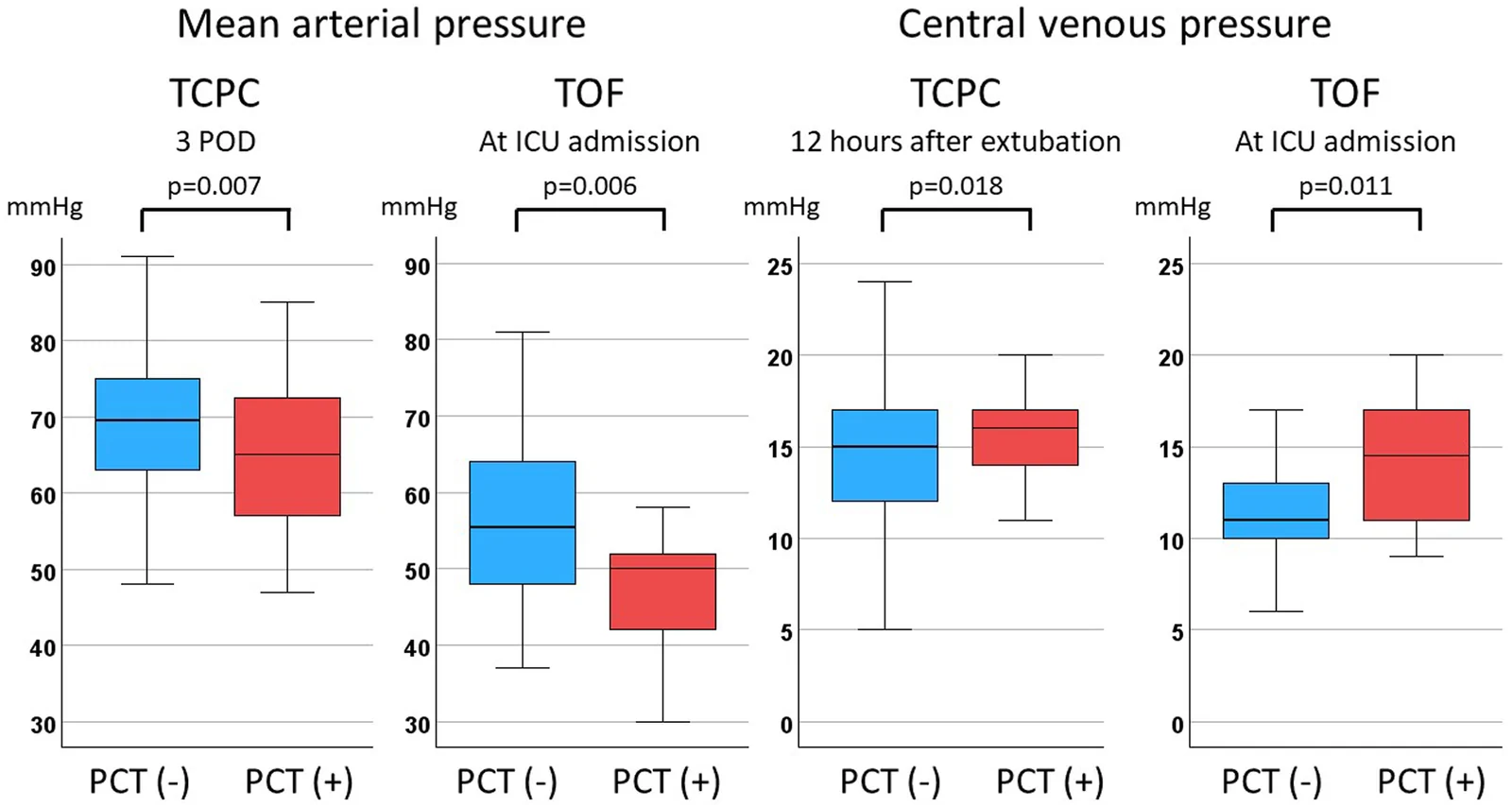
Box-and-whisker plots of mean arterial pressure and central venous pressure in patients with total cavopulmonary connection (*TCPC*) and tetralogy of Fallot (*TOF*). The upper and lower whiskers mark the minimum and maximum values, the *upper* and *lower borders* of the *box* represent the upper and lower quartiles, and the *middle horizontal line* represents the median. *POD*, Postoperative day; *ICU*, intensive care unit.

**Table 5:** Hemodynamic and laboratory variables in patients with TOF

Variable	PCT (+)		PCT (−)		*P* value
	Mean	SD	Mean	SD	
Mean arterial pressure					
At ICU admission	47.17	7.998	55.80	10.369	**.006**
6 h	51.00	8.279	56.28	9.969	.079
12 h	56.50	12.881	59.71	10.109	.310
24 h	58.42	8.785	62.37	10.669	.218
At ICU discharge	63.67	7.762	63.95	11.308	.934
Central venous pressure					
At ICU admission	14.25	3.415	11.62	3.364	**.011**
6 h	13.58	5.468	11.46	2.838	.210
12 h	13.42	4.944	11.36	3.290	.054
24 h	14.25	3.671	12.70	4.004	.202
At ICU discharge	10.50	3.782	12.78	3.664	.155
Arterial oxygen saturation					
At ICU admission	92.42	7.798	97.95	3.394	**.032**
6 h	96.33	3.143	98.11	2.557	**.027**
12 h	96.42	3.848	97.80	3.066	.150
24 h	96.92	2.712	97.13	2.895	.806
At ICU discharge	96.83	3.010	97.72	4.546	.513
Lactate levels					
At ICU admission	1.875	1.506	1.350	0.440	.254
6 h	2.033	1.017	1.665	0.699	.244
12 h	3.375	3.558	2.208	0.919	.281
24 h	2.617	1.438	1.885	1.504	.110
At ICU discharge	1.273	0.304	1.180	0.495	.547

Bold type indicates statistical significance. *TOF*, Tetralogy of Fallot; *PCT*, postoperative chylothorax; *SD*, standard deviation; *ICU*, intensive care unit.

### Treatment, Hospital Length of Stay, and Mortality

MCT was performed in 309 patients (99.0%), with a median duration of 92 days (IQR, 61-98 days). TPN was administered to 18 patients (5.8%) with a median duration of 15 days (IQR, 12-23 days). Octreotide therapy was administered to 28 patients (9.0%), with a median duration of 10 days (IQR, 9-14 days) and a median maximum dose of 15 μg/kg/hour. Additionally, a phosphodiesterase type 5 inhibitor (Sildenafil) was administered to 104 patients (33.3%), with a median dose of 12 (IQR, 9-21) mg/day. Other medical therapies included a beta-blocker (bisoprolol) in 83 patients (26.6%) and a corticosteroid (prednisolone) in 56 patients (17.9%). Anticoagulation with coumadin was provided to 152 patients (48.7%). Surgical treatment was performed in 11 patients (3.5%), with a median duration of 41 days (IQR, 18-71 days). Procedures included 3 pleurodeses and 2 thoracic duct ligations. Additionally, in patients after TCPC, 4 fenestrations were created, and 2 TCPC pathways were revised. Catheter interventions were performed in 51 patients (16.3%), with a median duration of 17 days (IQR, 11-24 days). Interventions included 25 closures of aortopulmonary collaterals, 12 stent implantations in the left pulmonary artery, 4 balloon dilatations of the superior vena cava/innominate vein, 3 balloon dilatations of the left pulmonary artery, 2 balloon dilatations of the TCPC pathway, and 1 stent implantation in the inferior vena cava–TCPC anastomosis. Two patients underwent MR lymphangiography, but no congenital lymphatic malformations were found. A superior vena cava thrombus was found in 15 patients (4.8%), all of whom were treated conservatively. The median hospital length of stay was longer in patients who developed PCT compared to those who did not (23 [IQR, 14-37] days vs 12 [IQR, 9-20] days; *P* < .001).

## Discussion

In this study, PCT following congenital heart surgery was observed in 3.2% of patients. The Fontan procedure was the most frequently performed procedure (20.5%), followed by TOF + AVSD repair (11.8%), ASO + VSD closure + aortic arch repair (10.0%), and TOF repair with a transannular patch (6.7%). Younger age at operation, TCPC, TOF + AVSD repair, ASO + VSD closure + aortic arch repair, TOF repair with transannular patch, and complete AVSD repair were identified as risk factors for PCT. Lower mean arterial pressure and higher central venous pressure were associated with the development of PCT in patients following TCPC and TOF repair. These results are not entirely novel, but our large cohort provides contemporary data on the incidence and risk factors for PCT, offering useful clinical information. See the Graphical Abstract for a visual summary or the Video 1 message for a verbal one.

### Incidence of PCT After Congenital Heart Surgery

Previous studies have reported an incidence of PCT after congenital heart surgery ranging from 1% to 9%.^1^, ^2^, ^3^, ^4^, ^5^^,^^9^ This wide range is due to heterogeneity in diagnoses, procedures, and surgical expertise. Recent studies suggest an increase in PCT prevalence from the previously reported 1% or less^3^, ^4^, ^5^, ^6^ to 4% to 9%,^4^^,^^7^^,^^8^^,^^18^^,^^19^ which has been attributed to the increasing complexity of surgical procedures and possibly earlier reintroduction of feeding after surgery.^18^^,^^19^ In the present study, the 3.2% incidence of PCT and higher incidence in the late era observed across a population spanning the full spectrum of procedures from preterm neonates to older adults over >20 years were consistent with the literature. The higher incidence of PCT in the late era may be attributed to younger age at operation and more complex procedures.

### Risk Factors for PCT

The risk of PCT has been reported to be highest in children undergoing the Fontan procedure and other complex procedures.^3^^,^^9^ Chan and colleagues^19^ demonstrated that patients undergoing cavopulmonary anastomosis were more likely to develop PCT. Mery and colleagues^10^ showed that the incidence of PCT increases with procedural complexity, as measured by the RACHS score. Biewer and colleagues^8^ found that complex procedures such as the Norwood procedure, AVSD repair, procedure for transposition of the great arteries, and truncus arteriosus repairs had PCT rates of 11% to 33%. Our results are consistent with those previous findings. We found the highest incidence of PCT in patients after the Fontan procedure. Specific hemodynamic features of Fontan circulation predispose to PCT development. Additionally, a high incidence of aortopulmonary collaterals and occasionally antegrade pulmonary blood flow can contribute to PCT.^20^ In this study, stage II BCPS was not identified as a risk factor for PCT. We usually perform BCPS on infants at age 3 to 6 months and close all antegrade pulmonary blood flow/shunt flow at the time of BCPS. In this situation, postoperative central venous pressure is lower than that after TCPC, which likely explains why stage II BCPS was not identified as a risk factor for PCT.

Younger age groups (neonates, infants, and young children) have been reported to have a significantly higher incidence of PCT than older age groups (children and teenagers).^8^^,^^10^^,^^19^ Our results also identified younger age as a significant risk for PCT. In this study, genetic anomalies, extracardiac anomalies, or anomalous systemic venous connections were not risk factors for PCT.

### Hemodynamic Effects on PCT

Mechanisms of PCT appear to be multifactorial, including hemodynamic disturbances, mechanical trauma to lymphatic vessels, and underlying congenital lymphatic malformations. Our results in TCPC and TOF patients showed higher central venous pressure and lower mean arterial pressure in patients with PCT compared to those without PCT. In this study, thoracic duct ligation was performed in only 2 patients, and no congenital lymphatic malformations were found. Therefore, our findings, along with other reports, suggest that early postoperative hemodynamic status is the main cause of PCT, and that other causes, such as mechanical trauma to lymphatic vessels or congenital lymphatic malformations, may be rare factors in the development of PCT. Procedures that elevate systemic venous pressure may be more likely to cause PCT.^3^

### Management of PCT

There are no definitive guidelines for the optimal management of PCT. As such, institutions have reported using different algorithms based on the physician's experience.^3^^,^^12^^,^^13^ A commonly adopted first-line approach is conservative management, including dietary modifications such as an MCT diet or nothing-by-mouth status with TPN, followed by medications. In our study, nearly all patients received dietary modifications with an MCT diet, whereas TPN was required in only 6%.

The choice of therapeutic strategies and the time elapsed before escalating to more invasive approaches are variable. Previous studies have suggested that prolonged chyle output (>2-3 weeks)^1^ and lesions associated with persistently elevated systemic venous pressure^4^^,^^7^ are key factors in decision making. For patients who do not respond to conservative management, more invasive approaches, including surgical interventions, such as pleurodesis, thoracic duct ligation, and pleuroperitoneal shunting, have been reported with variable success rates in pediatric patients, including those with refractory chylothorax.^4^^,^^7^ Fenestration creation in patients after the Fontan procedure also has been used to reduce elevated venous pressure.^18^^,^^21^

### Future Prospective

For nonresponders to conservative management, advanced therapies aimed at alleviating hemodynamic derangements require further investigation. Recent advances in imaging technologies for central lymphatics have improved our understanding of the pathophysiology of these conditions and may guide interventions to improve outcomes for these patients. Dori and colleagues demonstrated that dynamic contrast-enhanced MR lymphangiography allows assessment of abnormal lymphatic drainage and is the preferred method for evaluating chylothorax.^22^^,^^23^ They demonstrated the feasibility of various percutaneous interventions to restore normal lymphatic function.^4^^,^^24^ These options may improve PCT management strategies, including early diagnosis of PLPS or CLFD. In these patients, early selective lymphatic embolization of the pathologic lymphatic network, thoracic duct angioplasty, or lymphovenous anastomosis may be the first-line treatment options.

### Limitations

This study has several limitations, owing primarily to its single-center, retrospective design. Consequently, long-term follow-up data and comprehensive clinical information are unavailable, limiting our ability to fully evaluate clinical risk factors and perform adequate risk adjustment. Furthermore, changes in surgical and medical management over time might not be fully captured in our analyses and could impact the results. Thoracic duct ligation was performed twice in our cohort. It was used not as a primary treatment for suspected anatomic injury to the thoracic duct but rather as a palliative last-resort measure in patients with persistent PCT refractory to conservative management. Although our overall findings suggest that postoperative hemodynamics may play a predominant role in the development of PCT, exact mechanisms were not proven by lymphangiography.

## Conclusions

In this large-scale, single-center study, records of approximately 10,000 congenital heart surgeries were examined, and PCT was observed in 3.2% of the patients. The Fontan procedure had the highest incidence, followed by complex biventricular repair in neonates and young infants. A significant complication in pediatric cardiac surgery, PCT is associated with increased hospital length of stay and the need for additional treatment. Management strategies should be optimized using advanced technologies, such as MR lymphangiography and percutaneous interventions, to restore normal lymphatic function.

## Conflict of Interest Statement

The authors reported no conflicts of interest.

The *Journal* policy requires editors and reviewers to disclose conflicts of interest and to decline handling or reviewing manuscripts for which they may have a conflict of interest. The editors and reviewers of this article have no conflicts of interest.

## Figures and Tables

**Table E1:** Baseline and operative characteristics of patients by era

Variable	All patients	Early era	Late era	*P* value
Number of patients (%)	9892	5153 (52.1)	4739 (47.9)	
PCT, n (%)	312 (3.2)	106 (2.1)	206 (4.3)	**<.001**
Preoperative variables, n (%)				
Male sex	5567 (56.3)	2905 (56.4)	2662 (56.2)	.839
Genetic anomalies	1384 (14.5)	714 (14.8)	670 (14.2)	.420
Extracardiac anomalies	1721 (18.1)	896 (18.7)	825 (17.5)	.162
Heterotaxy syndrome	211 (2.2)	101 (2.1)	110 (2.3)	.446
Hypothyroidism	237 (2.5)	113 (2.4)	124 (2.6)	.378
Operative variables				
Age at procedure, y, median (IQR)	1.2 (0.3-9.8)	1.3 (0.3-9.6)	1.0 (0.3-9.9)	**<.001**
Weight at procedure, kg, median (IQR)	8.5 (4.5-25.6)	9.4 (4.8-24.1)	8.0 (4.4-27.0)	.533
OP in neonates, n (%)	1649 (16.7)	849 (16.5)	800 (16.9)	.589
OP in infants, n (%)	3125 (31.6)	1563 (30.3)	1562 (33.0)	**.005**
OP in young children (≥1 & <6 y), n (%)	2115 (21.4)	1149 (22.3)	966 (21.4)	**.022**
OP in older children (≥6 & <12 y), n (%)	789 (8.0)	483 (9.4)	306 (6.5)	**<.001**
OP in teens (≥12 & <18 y), n (%)	699 (7.1)	421 (8.2)	278 (5.9)	**<.001**
OP in adults (≥18 y), n (%)	1515 (15.3)	688 (13.4)	827 (17.5)	**<.001**
STAT category, n (%)				
1	4195 (42.4)	2229 (53.1)	1966 (41.5)	.075
2	2508 (25.4)	1289 (25.0)	1219 (25.7)	.419
3	1083 (10.9)	548 (10.6)	535 (11.3)	.297
4	1682 (17.0)	879 (17.1)	803 (16.9)	.881
5	424 (4.3)	208 (4.0)	216 (4.6)	.201
4 or 5	2106 (21.3)	1087 (21.1)	1019 (21.5)	.621
CPB time, min, median (IQR)	86 (55-127)	79 (50-118)	95 (61-137)	**<.001**
Need for aortic cross-clamp, n (%)	7278 (73.6)	3445 (66.9)	3833 (80.9)	**<.001**
Procedure, n (%)				
Norwood	408 (4.1)	199 (3.9)	209 (4.4)	.171
BCPS	586 (5.9)	312 (6.1)	274 (5.8)	.566
TCPC	584 (5.9)	318 (6.2)	266 (5.6)	.239
TOF AVSD repair	17 (0.2)	6 (0.1)	11 (0.2)	.165
ASO + VSD + arch	20 (0.2)	7 (0.1)	13 (0.3)	.126
TOF repair, TAP	240 (2.4)	121 (2.3)	119 (2.5)	.599
TAPVC repair	110 (1.1)	48 (0.9)	62 (1.3)	.074
Complete AVSD repair	392 (4.0)	184 (3.6)	208 (4.4)	**.037**
Postoperative variables				
Hospital deaths, n (%)	432 (4.4)	5 (0.2)	23 (0.8)	**.004**
Hospital length of stay, d	13 (9-20)	14 (10-21)	11 (7-18)	**<.001**

Bold type indicates statistical significance. *PCT*, Postoperative chylothorax; *IQR*, interquartile range; *OP*, operation; *STAT*, Society of Thoracic Surgeons–European Association for Cardio-Thoracic Surgery; *BCPS*, bidirectional cavopulmonary shunt; *TCPC*, total cavopulmonary connection; *TOF*, tetralogy of Fallot; *AVSD*, atrioventricular defect; *ASO*, arterial switch operation; *VSD*, ventricular septal defect; *TAP*, transannular patch; *TAPVC*, total anomalous pulmonary venous connection.

## Supplementary Data

Video 1 The author, Verena Kunke, briefly explains the study's importance and relevance. Video available at: https://www.jtcvs.org/article/S2666-2736(26)00485-7/fulltext.
